# Rethinking Breast Imaging Reporting and Data System (BI-RADS) 4: Clinical Insights From Mammographic Subclassification

**DOI:** 10.7759/cureus.94379

**Published:** 2025-10-12

**Authors:** Deepak Pradeep Kumar, Alex X Chakiath, Aneesh Sugunan, Kasthoory Kandiah, Rahi R Gandhi, Jim Job, Jasira Padinhare Madathil, Pradeep Chandran, Nandini Varma, Abhijith Mohan Rajamohan

**Affiliations:** 1 Radiology, Addenbrooke’s Hospital, Cambridge University Hospital, Cambridge, GBR; 2 Surgical Oncology, Ernakulam Medical Trust Hospital, Kochi, IND; 3 Breast and Endocrine Surgery, Believers Church Medical College Hospital, Thiruvalla, IND; 4 General Surgery, King's College Hospital, London, GBR; 5 General Surgery, Princess Royal University Hospital, London, GBR; 6 Breast and General Surgery, Homerton University Hospital, London, GBR; 7 Trauma Surgery, King's College Hospital, London, GBR; 8 Radiology, Mersey and West Lancashire Teaching Hospital, Liverpool, GBR; 9 Radiology, Bristol Royal Infirmary, Bristol, GBR

**Keywords:** bi-rads 4, breast cancer, breast imaging, breast imaging reporting and data system (bi-rads), breast malignancy, general surgery

## Abstract

Introduction: This study assesses the predictive value of clinical factors and Breast Imaging Reporting and Data System (BI-RADS) 4 subcategories in identifying malignant breast lesions. A retrospective review of women with BI-RADS 4 lesions undergoing biopsy evaluated histopathological outcomes in correlation with imaging subcategories and clinical parameters. The predictive performance of subclassification and relevant patient factors was analyzed to refine risk assessment for malignancy.

Methods: A review of 190 women with BI-RADS 4 lesions identified between January 2022 and August 2025 was performed. Palpable lesions underwent excision and frozen section, while non-palpable lesions had wire-guided biopsy. Histopathology was correlated with imaging subcategories and clinical variables. Statistical analyses included chi-square tests and logistic regression.

Results: Of 190 lesions, 136 (71.6%) were 4a, 32 (16.8%) were 4b, and 22 (11.6%) were 4c. Final histology revealed 49 invasive carcinomas (25.8%), nine (4.7%) ductal carcinoma in situ, and 132 (69.4%) benign lesions. Malignancy rates were 18.4% for 4a, 31.3% for 4b, and 63.6% for 4c. Non-palpable lesion malignancy increased with subcategory (16.7%, 38.9%, and 100%). Age >50 significantly predicted malignancy (35.5% vs. 16.5%, OR: 3.0, p = 0.003). Prior breast cancer history showed a negative association (OR: 3.3, p = 0.001), especially in contralateral lesions. Diagnostic validity improved with higher BI-RADS subcategories, with 4c showing the best specificity (94.3%) and accuracy (77.4%). Fibroadenoma was the most common benign diagnosis (62.8%).

Conclusion: Age over 50 years and prior breast cancer are strong independent predictors of malignancy in BI-RADS 4 lesions. Subcategories stratify risk and malignancy rates, especially in 4a, which exceeded expected ranges, underscoring limitations in current classification. Incorporating clinical history and radiomics into BI-RADS may refine risk prediction and reduce unnecessary biopsies.

## Introduction

The Breast Imaging Reporting and Data System (BI-RADS), described by the American College of Radiology, is a common acronym used for better understanding and communication of the abnormalities detected during breast imaging among treating physicians and radiologists. The use of BI-RADS is not limited to conventional mammograms, but also includes MRIs and ultrasounds as well [1]. BI-RADS 1, 2, and 3 deal with benign pathology, while BI-RADS 4 is reported when “there are findings that do not have the classic appearance of malignancy, but are sufficiently suspicious to justify the recommendation of a biopsy” [2]. BI-RADS 5 deals with “very high chances of malignancy”, that is, more than 95%, while BI-RADS 6 indicates a biopsy-proven malignancy.

BI-RADS 4 indicates a broad risk of malignancy, ranging from 2% to 95%, and is therefore further subdivided into three categories. BI-RADS 4a suggests low suspicion for malignancy (2-10%), BI-RADS 4b suggests moderate suspicion of malignancy (10-50%), and BI-RADS 4c suggests high suspicion of malignancy (50-95%) [2,3]. BI-RADS 4 findings can include irregular shapes, indistinct or microlobulated margins, nonparallel orientation, hypoechoic appearance on ultrasound, and amorphous or fine pleomorphic calcifications on mammography. It has been reported that the age of the patient is a significant risk factor for detecting malignancy in a BI-RADS 4 lesion and that younger age patients are at a lower risk [4]. This study aims to determine the positive predictive value of BI-RADS 4 and its subcategories in detecting malignancy. This study also aims to assess other factors, such as age and history of breast cancer, which may suggest a higher risk among the BI-RADS 4 category.

## Materials and methods

Study design

This retrospective observational study was conducted at our tertiary care hospital, focusing on all female patients who were evaluated for breast lesions classified as BI-RADS 4 on mammography or breast ultrasound between January 2022 and August 2025. The primary goal was to quantify the predictive value of BI-RADS 4 subcategories alongside select clinical variables for malignant histopathology. All patients included in the study underwent image-guided or open biopsy, with subsequent histopathological analysis serving as the reference standard.

Beyond its primary aim of assessing the predictive value of BI-RADS 4 subclassification and clinical factors in malignancy detection, this study explored several secondary objectives pertinent to breast lesion evaluation. Notably, the research evaluated patterns of biopsy utilization in palpable versus non-palpable lesions and highlighted the tendency for wire-guided biopsies to yield more benign results, underlying potential clinical decision biases and the diagnostic challenges with non-palpable findings. The investigation also probed the diagnostic validity metrics, such as sensitivity, specificity, and likelihood ratios, for each BI-RADS 4 subcategory, revealing marked discrepancies between expected and observed malignancy rates, especially for 4a lesions. Moreover, the study analyzed the effect of age and previous breast cancer history on malignancy risk, offering evidence that advanced age and contralateral history substantially elevate risk, while ipsilateral history does not necessarily predict subsequent malignancy, which could inform future surveillance strategies. The distribution of benign histology, particularly the predominance of fibroadenoma, and the balance of lesion laterality were also addressed, enriching the understanding of the broader clinicopathological spectrum encountered in BI-RADS 4 workup.

Inclusion and exclusion criteria

Eligible participants comprised female patients aged 18 years or older who presented with a breast lesion categorized as BI-RADS 4a, 4b, or 4c. It was mandatory that patients had completed a biopsy, with either wire-guided localization or excision for palpable/non-palpable lesions as appropriate, and had a finalized histopathology report available. Male patients, those lacking histopathology results, or individuals with a history of breast cancer without a current BI-RADS 4 lesion on imaging were excluded from the study. This strict inclusion framework ensured a focused evaluation of patients at risk for malignancy who met both imaging and pathological criteria for analysis.

Data collection

Detailed demographic data, including patient age, presenting symptoms, side and location of the breast lesion, and previous history of breast malignancy, were systematically recorded from electronic medical records. The biopsy approach was selected based upon clinical palpability: lesions that were palpable underwent skin marking and wide excision with intraoperative frozen section analysis, while non-palpable lesions were localized using wire guidance and then subjected to core or excision biopsy under imaging guidance. All biopsy tissue samples were then processed by institutional pathology services, with histopathological diagnoses reviewed for correlation with BI-RADS subcategory and clinical features.

Statistical analysis

Continuous variables were summarized using mean and standard deviation, while categorical variables were represented as frequencies and percentages. Group differences were assessed using the chi-square or Fisher’s exact test for categorical data. Binary logistic regression was implemented to evaluate the association between clinical risk factors (such as age above 50 years or prior breast cancer) and malignant histopathology, including calculation of odds ratios (ORs) and corresponding 95% confidence intervals (CIs). All analyses were performed using standard statistical software, with a p-value of less than 0.05 considered statistically significant.

Tool licensing

The BI-RADS used for categorization in this study is a standardized and internationally recognized clinical system developed by the American College of Radiology. BI-RADS is licensed under the Creative Commons Attribution-NoDerivatives 4.0 International License (CC BY-ND 4.0), permitting free use for non-commercial academic applications, provided appropriate credit is given and no modifications are made [5].

No other proprietary scales, scoring, or pathology tools requiring a separate license were employed in this study. All references to BI-RADS within tables and figures should include the above citation as a footnote or within legends, and the Methods section should clearly state the BI-RADS licensing status and citation. The histopathological reporting adhered to widely accepted clinical practice standards, which do not require special licensing.

## Results

As shown in Table 1, 190 patients underwent a biopsy following a BI-RADS 4 report on their mammogram. We had no male patients in this cohort. The mean age of the patients was 50.8 years, with a standard deviation of 10.6. A total of 97 patients were below the age of 50 years. A total of 94 of the BI-RADS 4 lesions were detected in the left breast, and the remaining in the right breast. A total of 136 patients had BI-RADS 4a lesions, 32 patients had BI-RADS 4b lesions, while 22 patients had BI-RADS 4c lesions. A total of 102 BI-RADS 4a lesions, 18 BI-RADS 4b lesions, and five BI-RADS 4c lesions were non-palpable. These 125 lesions underwent wire-guided biopsy. Among the 125 non-palpable lesions, 29 were reported as malignant. When only non-palpable lesions were considered, the risk of malignancy in BI-RADS 4a, 4b, and 4c was 16.67%, 38.89%, and 100%, respectively.

**Table 1:** Patient demographics. The Breast Imaging Reporting and Data System (BI-RADS) classifications used in this table are described by the American College of Radiology [5].

Variable	N (%) or mean ± SD
Total patients	190 (100)
Sex (female)	190 (100)
Mean age	50.8 ± 10.6
Patients <50 years	97 (51.1)
Patients ≥50 years	93 (48.9)
Left-sided lesions	94 (49.5)
Right-sided lesions	96 (50.5)
BI-RADS 4a	136 (71.6)
BI-RADS 4b	32 (16.8)
BI-RADS 4c	22 (11.6)
Non-palpable lesions	125 (65.8)
History of breast cancer	41 (21.6)
Contralateral history	32 (16.8)
Ipsilateral history	9 (4.7)
Fibroadenoma	83 (62.8 of benign cases)

Table 2 examines the relationship between wire-guided biopsy procedures and histopathological outcomes in BI-RADS 4a lesions. When analyzing non-palpable lesions specifically for BI-RADS 4a lesions, the data revealed a statistically significant association (p = 0.002, χ² = 9.577). Of the patients who did not undergo wire-guided biopsy for 4a lesions, 56 (39.7%) cases were benign and 32 (65.3%) cases were malignant, totaling 88 (46.3%) patients. Conversely, among patients who did receive wire-guided biopsy for 4a lesions, 85 (60.3%) cases were benign while only 17 (34.7%) cases proved malignant, comprising 102 (53.7%) patients. This distribution suggests that wire-guided biopsy was more commonly performed in cases that ultimately proved to be benign, which may reflect clinical decision-making patterns or the nature of non-palpable lesions requiring guided biopsy.

**Table 2:** Non-palpable BI-RADS 4a lesion distribution by histopathology reports. The Breast Imaging Reporting and Data System (BI-RADS) classifications used in this table are described by the American College of Radiology [5].

Non-palpable BI-RADS 4a	Histopathology report				Total		χ2	df	p
	Benign		Malignant						
	N	%	N	%	N	%			
No	56	39.7	32	65.3	88	46.3	9.577	1	0.002
Yes	85	60.3	17	34.7	102	53.7			
Total	141	100.0	49	100.0	190	100.0			

On following up the histopathology report (HPR), 49 (25.8%) patients among the 190 had invasive breast carcinoma, nine had ductal carcinoma in situ, and the remaining 132 had benign pathologies. Among the BI-RADS 4a lesions, 25 were malignant, which shows a risk of malignancy of 18.38%. Among the remaining 24 malignant pathologies, 10 were reported as B-IRADS 4b and 14 were reported as BI-RADS 4c, attributing a risk of malignancy of 31.25% and 63.64%, respectively, to these groups. Tables 3-5 examine the diagnostic validity of BI-RADS 4a, 4b, and 4c, respectively.

**Table 3:** Diagnostic validity of BI-RADS 4a lesions. PPV: positive predictive value; NPV: negative predictive value; LR: likelihood ratio. The Breast Imaging Reporting and Data System (BI-RADS) classifications used in this table are described by the American College of Radiology [5].

Parameter	Value
Sensitivity	51.0
Specificity	21.3
PPV	18.4
NPV	55.6
Accuracy	28.9
LR+	0.6
LR-	2.30

**Table 4:** Diagnostic validity of BI-RADS 4b lesions. PPV: positive predictive value; NPV: negative predictive value; LR: likelihood ratio. The Breast Imaging Reporting and Data System (BI-RADS) classifications used in this table are described by the American College of Radiology [5].

Parameter	Value
Sensitivity	20.4
Specificity	84.4
PPV	31.3
NPV	75.3
Accuracy	67.9
LR+	1.3
LR-	0.94

**Table 5:** Diagnostic validity of BI-RADS 4c lesions. PPV: positive predictive value; NPV: negative predictive value; LR: likelihood ratio. For the likelihood ratios (LR+ and LR−) shown in the table for BI-RADS 4c lesions, the hypotheses are as follows: Null hypothesis (H₀): The BI-RADS 4c test does not differentiate between malignant and benign lesions; that is, the likelihood ratio is equal to 1 (LR = 1), so the test result is equally likely in both groups. Alternate hypothesis (H₁): The BI-RADS 4c test does differentiate between malignant and benign lesions; that is, LR+ > 1 (for a positive test) and/or LR− < 1 (for a negative test), showing that the test modifies the probability of malignancy beyond chance. The Breast Imaging Reporting and Data System (BI-RADS) classifications used in this table are described by the American College of Radiology [5].

Parameter	Value
Sensitivity	28.6
Specificity	94.3
PPV	63.6
NPV	79.2
Accuracy	77.4
LR+	5.0
LR-	0.76

There were 16 invasive malignancies reported in patients less than or equal to 50 years of age, and 33 in patients above the age of 50. In patients below the age of 50 years, the risk of malignancy attributed to BI-RADS 4 overall, 4a, 4b, and 4c was 16.5%, 12.9%, 17.6%, and 40%, respectively. In patients above the age of 50 years, the risk of malignancy attributed to BI-RADS 4 overall, 4a, 4b, and 4c was 35.5%, 24.24%, 46.67%, and 83.34%, respectively.

As shown in Table 6, the relationship between age and malignancy shows a statistically significant association. Among patients under 50 years, 81 (83.5%) had benign lesions and only 16 (16.5%) had malignant lesions. In contrast, patients 50 years and older showed a higher malignancy rate, with 60 (64.5%) having benign lesions and 33 (35.5%) having malignant lesions. The chi-square test demonstrated a significant association (χ² = 8.945, p = 0.003), confirming that older patients have a significantly higher risk of malignancy in BI-RADS 4 lesions, with the malignancy rate more than doubling from 16.5% in younger patients to 35.5% in older patients.

**Table 6:** Significance of age in predicting malignancy in BI-RADS 4 lesions. HPR: histopathology report. The Breast Imaging Reporting and Data System (BI-RADS) classifications used in this table are described by the American College of Radiology [5].

HPR	Age				Total		χ2	df	p
	<50 years		>50 years						
	N	%	N	%	N	%			
Benign	81	83.5	60	64.5	141	74.2	8.945	1	0.003
Malignant	16	16.5	33	35.5	49	25.8			
Total	97	100.0	93	100.0	190	100.0			

The first logistic regression model (Table 7) examined age (>50 years) and BI-RADS 4a classification as independent predictors of malignancy. This analysis revealed that age greater than 50 years is a significant positive predictor of malignancy (p = 0.003, Wald = 8.93), with an adjusted odds ratio of 3.0 (95% CI: 1.5-6.1), indicating that patients over 50 years have a three times higher chance of having malignant lesions compared to younger patients with a BI-RADS 4a lesion.

**Table 7:** Binary logistic regression model: age and BI-RADS 4a as predictors of malignancy. The Breast Imaging Reporting and Data System (BI-RADS) classifications used in this table are described by the American College of Radiology [5].

	B	S.E.	Wald	df	p	Adj. OR	95% CI for adj. OR
Age >50 years	1.091	0.37	8.93	1	0.003	3.0	1.5 - 6.1
BI-RADS 4a	-1.33	0.37	13.19	1	0.000	0.3	0.1 - 0.5
Constant	-0.79	0.34	5.21	1	0.022	0.5	

A total of 41 (21.5%) patients had a history of breast cancer in the past. Among them, 32 (16.8%) had a history of breast carcinoma on the contralateral side, while nine (4.7%) developed lesions on the ipsilateral breast during the follow-up after breast conservation therapy. Of the 41 patients, 12 (29.26%) were found to have malignancy in the final HPR. None of those patients who were previously treated with breast conservation therapy, who were detected to have a BI-RADS 4 lesion in the mammogram, had malignancy in the final HPR, irrespective of BI-RADS 4a (four patients), 4b (three patients), and 4c (two patients) status. Among the patients with a history of contralateral breast carcinoma, 26 patients were detected to have BI-RADS 4a lesions, while four patients had BI-RADS 4b, and two patients had BI-RADS 4c lesions. The risk of malignancy in these categories was noted to be 30.77%, 50%, and 100%, respectively.

**Figure 1:**
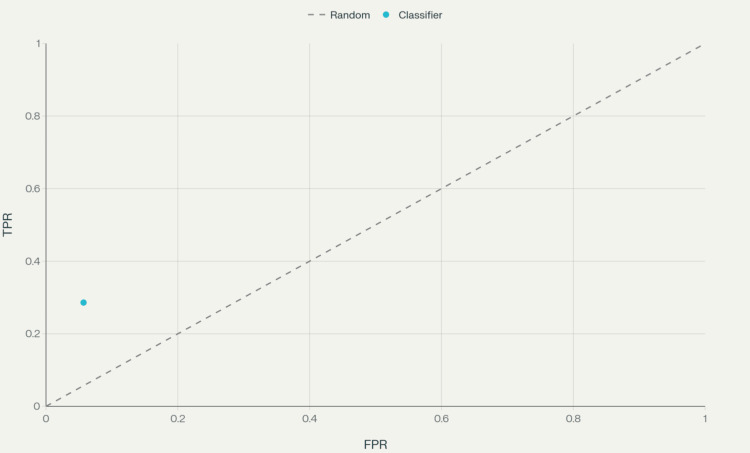
ROC curve for BI-RADS 4c logistic regression model (single point based on sensitivity and specificity). ROC curve for the binary logistic regression model parameters shown in Table 8, plotted using the sensitivity (28.6%) and specificity (94.3%) values. The single point represents the classifier’s true positive rate (TPR) vs. false positive rate (FPR). BI-RADS: Breast Imaging Reporting and Data System; ROC: receiver operating characteristic.

As shown in Table 8, patients with previous breast cancer with 4a lesions detected on follow-up showed a significant negative association with malignancy (p = 0.001, Wald = 11.32), with an adjusted OR of 0.3 (95% CI: 0.1-0.6), indicating that biopsies in the 4a category are less likely to be malignant. This finding is clinically significant as a personal history of breast cancer increases the risk of developing cancer in the opposite breast and may influence the interpretation of suspicious findings. Figure 1 shows the receiver operating characteristic (ROC) curve for Table 8.

**Table 8:** Binary logistic regression model: previous breast cancer history and BI-RADS 4a. The Breast Imaging Reporting and Data System (BI-RADS) classifications used in this table are described by the American College of Radiology [5].

	B	S.E.	Wald	df	p	Adj. OR	95% CI for adj. OR
Previous breast cancer	1.204	0.37	10.72	1	0.001	3.3	1.6 - 6.8
BI-RADS 4a	-1.23	0.36	11.32	1	0.001	0.3	0.1 - 0.6
Constant	-1.14	0.30	14.45	1	0.000	0.3	

The most common benign histology was fibroadenoma, which was noted in 83 (62.8%) patients. The rest included fibroadenosis, fibrocystic disease, benign phyllodes tumor, and chronic inflammatory changes.

## Discussion

In this study, the balanced age distribution is particularly relevant, given that age is identified as a significant risk factor for detecting malignancy in BI-RADS 4 lesions, with younger patients typically at lower risk. The balanced bilateral distribution suggests that there is no inherent predisposition for BI-RADS 4 lesions to occur more frequently on either side, which is consistent with general breast pathology patterns.

Among patients not classified as BI-RADS 4a, 30 (21.3%) cases were benign, while 24 (49.0%) cases were malignant, totaling 54 (28.4%) patients. In contrast, among those classified as BI-RADS 4a, 111 (78.7%) cases proved benign, and 25 (51.0%) cases were malignant, comprising 136 (71.6%) patients. This distribution reflects the clinical reality where most suspicious lesions fall into the lower suspicion category, requiring careful evaluation to distinguish between benign and malignant pathology.

The data demonstrate that while BI-RADS 4a classification was intended to identify lesions with low suspicion for malignancy (2-10%), the actual malignancy rate in this category was approximately 18.4% (25/136), which exceeds the expected range and suggests the need for careful evaluation of diagnostic criteria [1].

Non-palpable lesions in which wire-guided biopsy was more commonly performed were ultimately proved to be benign, which may reflect clinical decision-making patterns. This indicates that there is a need for better delineation of the nature of non-palpable lesions requiring guided biopsy.

The diagnostic performance for BI-RADS 4a lesions demonstrates relatively poor discriminatory ability. The diagnostic performance for BI-RADS 4b lesions shows improved specificity compared to 4a. The negative predictive value was 75.3%, with an overall accuracy of 67.9%. The likelihood ratios (LR+ = 1.3, LR- = 0.94) suggest moderate diagnostic utility. The diagnostic performance for BI-RADS 4c lesions demonstrates the best performance among all subcategories. The likelihood ratios (LR+ = 5.0, LR- = 0.76) indicate good diagnostic utility, particularly for ruling in malignancy.

BI-RADS 4 category was subclassified into 4a, 4b, and 4c with increasing predictive value for malignancy between 2% and 95% based on the lesion orientation, shape, boundary, margins, posterior acoustic shadowing, echogenic pattern, calcific distribution, and mass density [6]. BI-RADS 4 lexicon is used when the radiologist has an index of suspicion of malignancy but cannot confirm without a biopsy. The cancer-to-biopsy yield is noted to be around 30% [7]. Invasive procedure such as a trucut biopsy or an open biopsy adds to the cost and anxiety to the patient [8,9]. It was mentioned in a study by Flowers et al. that the number of biopsies can be reduced if biopsies are performed only if the risk of malignancy is more than 10% in imaging [7]. Although there is some concurrence amongst experienced radiologists in reporting these subcategories, it is not without major discrepancies [10]. Adding to this difficulty is the lack of rigid recommendations to subcategorize BI-RADS 4 lesions [11]. There have been attempts to reduce the number of biopsies by suggesting confounding factors like age, shape, and size of the lesion to BI-RADS 4 subcategories, but it is not without missing the diagnosis of invasive carcinoma [12,13]. Finding a lower-risk BI-RADS 4a, with less than 2% likelihood of malignancy, and suggesting short-term follow-up with serial ultrasound, comes with the flaw that the risk of malignancy is below the American College of Radiology-recommended risk for a BI-RADS 4a category. Also, this adds to further subcategorizing without addressing the patient's anxiety or cost-benefit ratio [4]. The current guidelines still recommend biopsy for an image-detected BI-RADS 4a lesion [14]. With advancements in imaging, conventional subcategorization into BI-RADS 4a, 4b, and 4c appears to be futile in preventing biopsy. The data from the institutes catering to the management of breast lesions should be accumulated to develop an individualized radiomics nomogram, which may benefit in avoiding unnecessary biopsies [15,16].

The principal limitations of this study stem from the single-center design, the relatively modest sample size (n = 190), and reliance on the opinion of a single radiologist for imaging interpretation. These constraints may affect the generalizability of results, introduce selection and reporting biases, and limit reproducibility for broader clinical settings.

## Conclusions

The findings of this study underscore that patient age, particularly age over 50 years, and a prior history of breast cancer are dominant predictors of malignant potential in BI-RADS 4 lesions. The use of BI-RADS subcategories revealed that malignancy rates often exceed anticipated ranges, especially for 4a lesions, highlighting the need for further multi-centric studies with a larger sample size to clarify the existing classification systems. Integration of clinical history and advanced imaging analytics may facilitate more precise risk stratification, reduce unnecessary biopsies, and ultimately enhance patient care. Continued accumulation of institutional data and incorporating radiomics into evaluation protocols could offer meaningful improvements to clinical outcomes for patients undergoing assessment of BI-RADS 4 lesions.
